# Supplementary material for: Superb Li-Ion Storage of Sn-Based Anode Assisted by Conductive Hybrid Buffering Matrix
Source: Nanomaterials (Basel). 2023 Oct 13;13(20):2757. doi: 10.3390/nano13202757 (PMC10609529; doi:10.3390/nano13202757)
Supplement: Supplementary file 1 [file nanomaterials-13-02757-s001.zip › nanomaterials-2647217-supplementary.pdf]

Supplementary Materials

Superb Li-ion storage of Sn-based anode assisted by the conductive hybrid buffering matrix

Jinsil Shin[1], Sung-Hoon Park[2*], Jaehyun Hur[1*]

[1]Department of Chemical and Biological Engineering, Gachon University, 1342 Seongnam-daero, Seongnam 13120, Republic of Korea

[2]Department of Mechanical Engineering, Soongsil University, 369 Sangdo-ro, Dongjakgu, Seoul 06978, Republic of Korea

Correspondence should be addressed to Prof. Jaehyun Hur* (jhhur@gachon.ac.kr) and Prof. Sung-Hoon Park* (leopark@ssu.ac.kr).

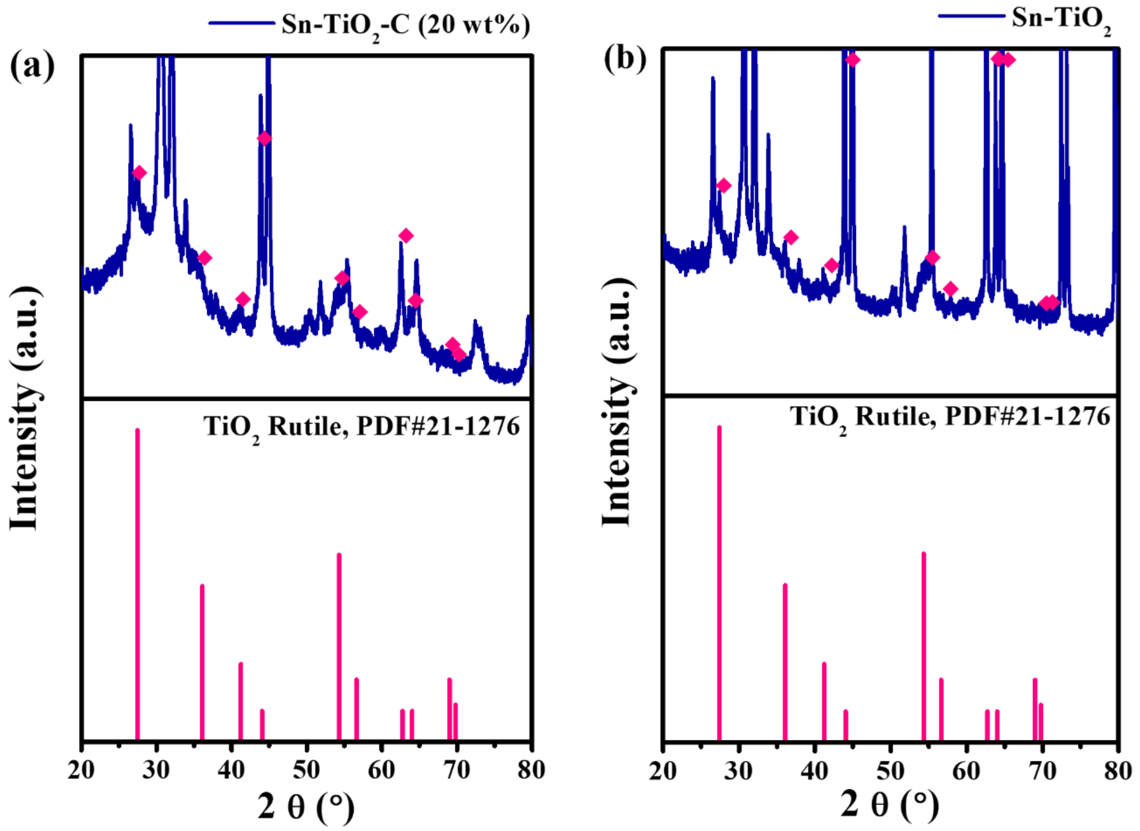

Figure S1. Enlarged view of XRD spectra of (a) Sn-TiO_2-C (20 wt%) and (b) Sn-TiO_2 along with theoretical peak of rutile TiO_2 (PDF#21-1276).

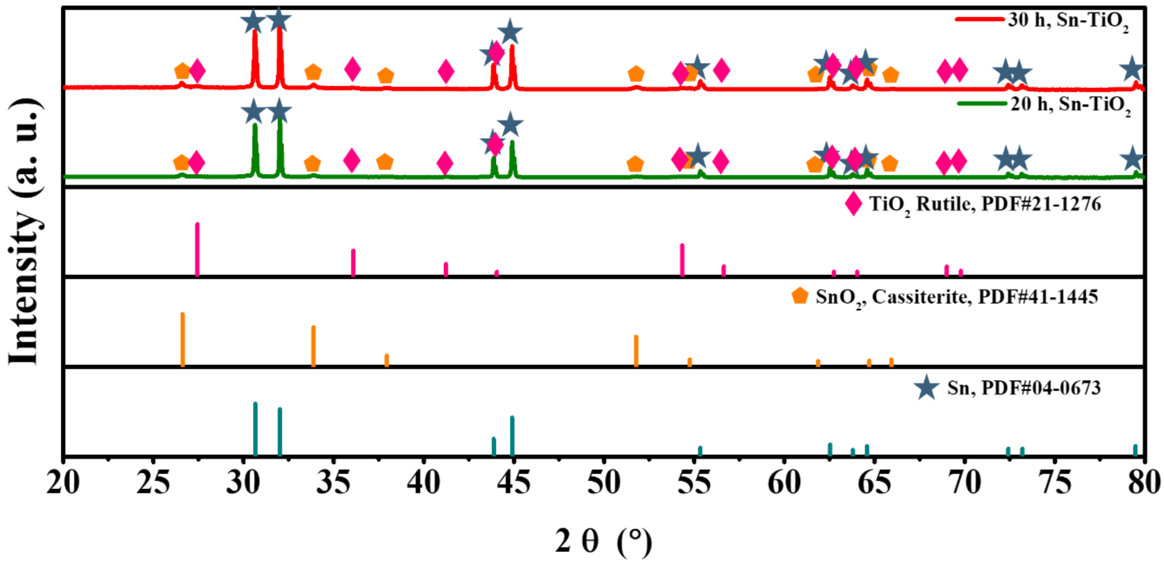

Figure S2. XRD spectra of Sn-TiO_2 after 20 h and 30 h milling.

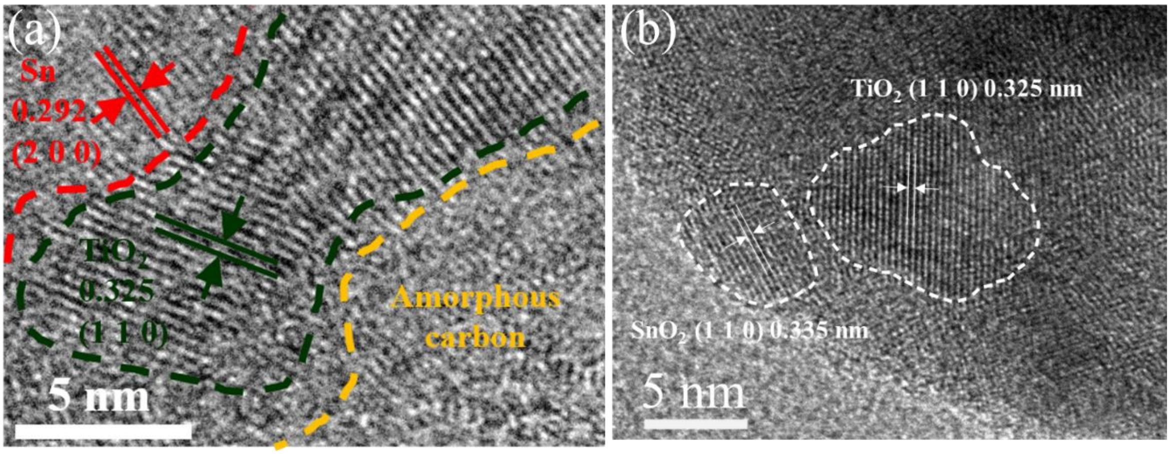

Figure S3. HRTEM image of Sn-TiO_2-C (20 wt%) showing the existence of (a) Sn, TiO_2 and amorphous carbon, (b) SnO_2.

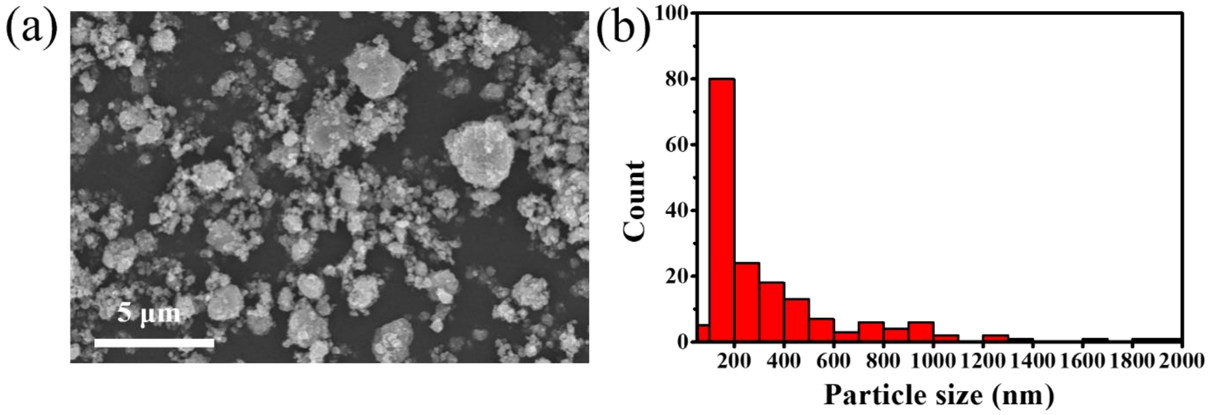

Figure S4. (a) SEM image and (b) particle size distribution of Sn-TiO_2-C (20wt%).

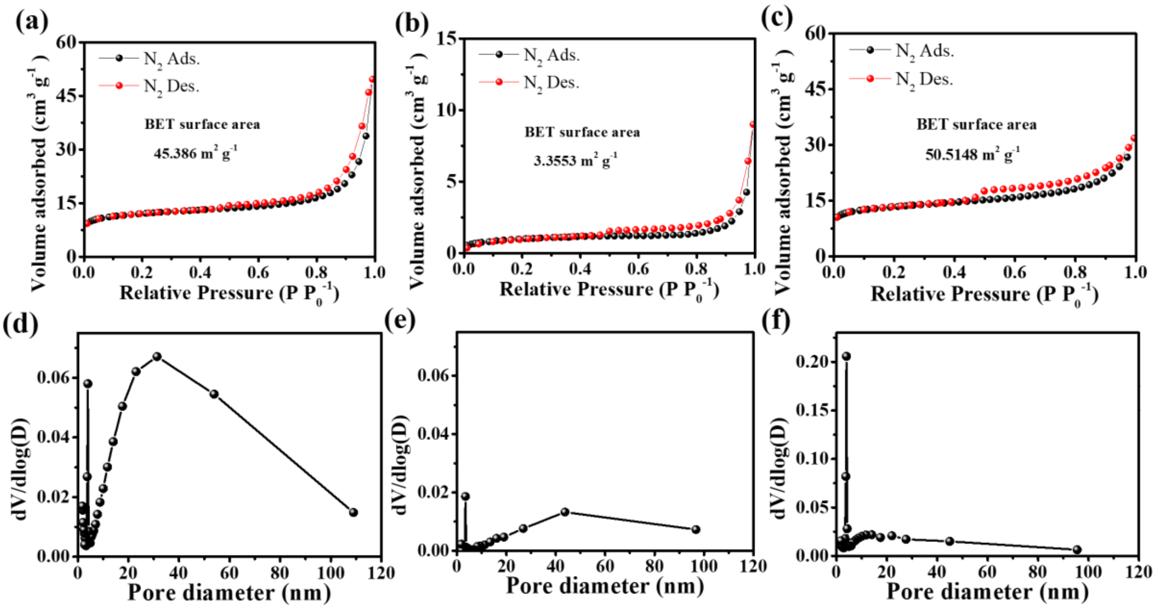

Figure S5. N_2 adsorption/desorption isotherm of (a) Sn-TiO_2-C (20 wt%), (b) Sn-TiO_2, and (c) Sn-C (20 wt%) at standard temperature and pressure condition. Pore distribution of (d) Sn-TiO_2-C (20 wt%), (e) Sn-TiO_2, and (f) Sn-C (20 wt%).

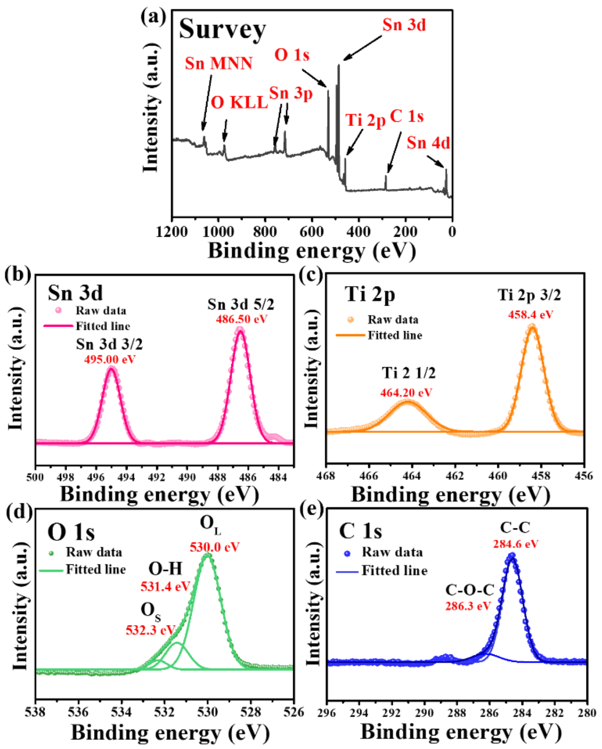

Figure S6. High-resolution XPS spectra of Sn-TiO_2 powder. (a) survey (b) Sn, (c) Ti, (d) O (O_L (lattice oxygen), O-H bond, and O_s (adsorbed oxygen)), and (e) C

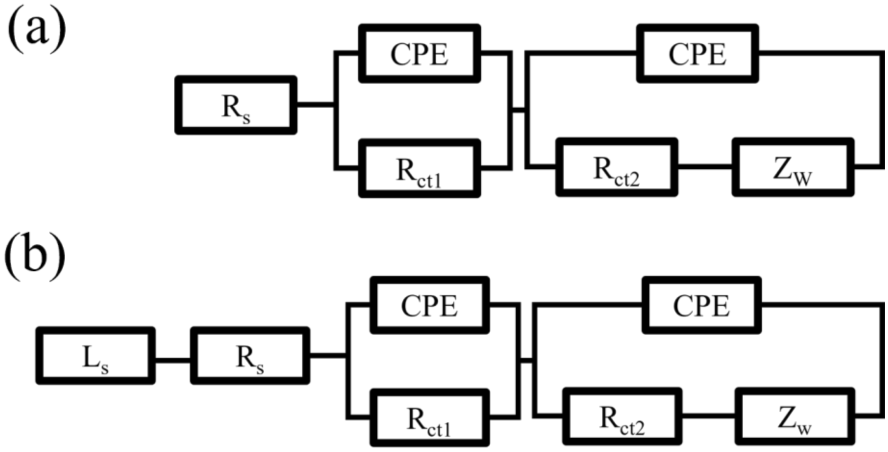

Figure S7. The equivalent circuit for (a) Sn-TiO_2-C (20 wt%) and (b) Sn-TiO_2 and Sn-C (20 wt%).

Table. S1. Theoretical capacity of Sn-TiO_2-C (20 wt%), Sn-TiO_2, and Sn-C (20 wt%)

Composite	Theoretical capacity
Sn-TiO_2-C (20 wt%)	657 mAh g^{-1}
Sn-TiO_2	728 mAh g^{-1}
Sn-C (20 wt%)	869 mAh g^{-1}

Table S2. Performance of Sn-based anode for Li-ion batteries

Composite	Specific capacity	Cycles number	Current density	Ref
Sn-C	410 mAh g^{-1}	100 cycles	100 mA g^{-1}	[19]
Sn-Co	515.5 mAh g^{-1}	50 cycles	50 mA g^{-1}	[20]
Sn/CNT	413 mAh g^{-1}	100 cycles	30 mA g^{-1}	[21]
Sn-Ca	280 mAh g^{-1}	60 cycles	50 mA g^{-1}	[22]
3D Cu-Sn	590 mAh g^{-1}	50 cycles	0.3 C	[23]

3D DHP Cu-Sn	11.95 mAh cm^{-2}	50 cycles	1 mA cm^{-2}	[24]
Np-$GeSn_5$	974mAh g^{-1}	500 cycles	200 mA g^{-1}	[25]
Sn-MOF/G	462 mAh g^{-1}	500 cycles	1 A g^{-1}	[26]
Sn-Ni	448.9 mAh g^{-1}	20 cycles	0.1 C	[27]
SnSb-C	300 mAh g^{-1}	100 cycles	100 mA g^{-1}	[28]
SnS_x/NRGO	562 mAh g^{-1}	200 cycles	0.2 A g^{-1}	[29]
Sn-TiO_2-C	669 mAh g^{-1}	100 cycles	200 mA g^{-1}	This work

Table S3. Coulombic efficiency of Sn-TiO_2-C (20 wt%), Sn-TiO_2, and Sn-C (20 wt%) at 1, 2, 3, 10, and 50th cycle at current density of 200 mA g^{-1}

**Cycle number**	**Coulombic efficiency**		
	Sn-TiO_2-C (20 wt%)	Sn-TiO_2	Sn-C (20 wt%)
**1st**	81.89	63.54	73.50
**2nd**	84.38	67.56	90.78
**3rd**	95.51	89.12	91.24
**10th**	98.31	96.19	94.43
**50th**	98.73	98.30	97.54

Table S4. Fitted parameter values of R_s, R_{ct1}, and R_{ct2} of Sn-TiO_2-C (20 wt%), Sn-TiO_2, and Sn-C (20 wt%) at 200 mA g^{-1} after 20 cycles

Parameter	Sn-TiO_2-C (20 wt%)	Sn-TiO_2	Sn-C (20 wt%)
R_s	2.62	5.59	3.56
R_{ct1}	4.83×10^1	6.45×10^1	9.83×10^1
R_{ct2}	1.80×10^2	2.17×10^2	1.95×10^2
